# Development and preliminary evaluation of the QUALIKO: an observational quality of life instrument for patients with Korsakoff’s syndrome

**DOI:** 10.1186/s12955-020-01463-4

**Published:** 2020-07-21

**Authors:** Peter M. ten Klooster, Yvonne C. M. Rensen, Jorrit F. Postma, Roy P. C. Kessels

**Affiliations:** 1 Department of Psychology, Health, and Technology, University of Twente, Enschede, The Netherlands; 2 Center of Excellence for Korsakoff and Alcohol-Related Cognitive Disorders, Vincent van Gogh Institute for Psychiatry, Venray, The Netherlands; 3 ZorgAccent, Hellendoorn, The Netherlands; 4 Donders Institute for Brain, Cognition and Behaviour, Radboud University, PO Box 9104, 6500 HE Nijmegen, The Netherlands; 5 Department of Medical Psychology, Radboud University Medical Center, Nijmegen, The Netherlands

**Keywords:** Korsakoff’s syndrome, Nursing homes, Psychometrics, Quality of life

## Abstract

**Background:**

To develop a Korsakoff-specific measure of quality of life (QoL), to be rated by professional caregivers, and to field-test its psychometric properties in a sample of patients with Korsakoff’s syndrome (KS) living in a specialized nursing home.

**Methods:**

A research version of the QUALIKO was developed based on an existing instrument for dementia (the QUALIDEM), literature review and two rounds of surveys among expert professionals involved in the care for patients with KS. Next, QoL was independently rated using the preliminary QUALIKO for 77 patients with KS by two primary caregivers.

**Results:**

The research QUALIKO consisted of 48 items describing observable behaviors across ten aspects of QoL relevant to patients with KS. Six items demonstrated poor scalability in the field test. The remaining 42 items all formed subscales with moderate to strong scalability according to Mokken scale analysis. Reliability was acceptable to good across both raters for all subscales (Mokken rho’s = 0.70–0.90), except for the two 2-item subscales of negative affect and positive self-image (Mokken rho’s = 0.47–0.71). Inter-observer agreement was excellent for five subscales (ICCs = 0.75–0.89) and fair to moderate for the other five subscales (ICCs = 0.59–0.72). The multidimensional internal structure was confirmed and all subscales were significantly correlated with primary caregivers’ global ratings of QoL except for positive self-image. Missing item values were low and floor and ceiling effects acceptable for most subscales.

**Conclusions:**

The QUALIKO holds promise as a feasible, reliable, and valid measure of QoL in residential KS patients. Future research in larger samples is needed to confirm the psychometric dimensionality of the instrument, to gather normative data and to examine its test-retest reliability.

## Background

Korsakoff’s syndrome (KS) is a largely irreversible residual syndrome, typically resulting from severe nutritional (thiamine) depletion and occurring after incomplete recovery from a Wernicke encephalopathy [1]. KS occurs in most cases in chronic patients with Alcohol-Use Disorder and malnutrition. It is characterized by disproportionate learning and memory impairments [2, 3], executive dysfunction, flattened affect, apathy, lack of illness insight, and confabulations [1]. Due to these severe cognitive impairments and neuropsychiatric symptoms, most patients with KS are in need of lifelong specialized care. In the Netherlands, many KS patients reside in long-term care facilities from a relatively young age (mean age of admission of 56.7 [4]).

Given the severity of the symptoms associated with KS and the major impact these symptoms have on the long-term functioning and daily lives of patients with KS, gaining insight into the quality of life (QoL) of individual residential patients with KS is essential. In addition, due to a lack of illness-insight, patients with KS may become frustrated, suspicious, angry, and aggressive in long-term care settings, as they may not fully comprehend why they are unable to live independently in their own homes [5]. However, assessment of QoL of patients with KS living in long-term care facilities has to date received limited attention and instruments are lacking.

QoL is by definition subjective in nature and therefore preferably assessed using self-report questionnaires [6]. However, KS severely affects the cognitive functions of patients, resulting in a lack insight into oneself and one’s disease [7–9]. Because of this lack of illness insight and memory dysfunction, QoL of patients with KS is probably more reliably and validly assessed indirectly by using observational proxy instruments [6]. To date, no validated instruments for assessing QoL of patients with KS living in residential settings are available. By lack of a better fitting instrument, previous studies examining QoL in patients with KS used the QUALIDEM [10–14]. This is an observation scale developed for objectifying QoL in patients with dementia in nursing homes. The scale contains 37 questions that can be divided into nine subscales: Caregiver relationship, positive affect, negative affect, restless behavior, positive self-image, social relations, social isolation, feeling at home, and having something to do. For instance, Oudman and Zwart [12] compared the QoL of patients with KS and patients with dementia, both living in long-term care facilities, using the QUALIDEM. Overall, they found that QoL was higher in patients with KS than in dementia patients. However, the mean QoL score in the patients with KS could be considered moderate. The lowest scores were found on subscales “care relationship” and “having something to do”. Furthermore, patients felt less at home in a nursing home than patients with dementia. These scores on QoL remained relatively stable over a 20-month period [13].

Although patients with (Alzheimer’s) dementia and KS both display cognitive and memory deficits [15], the concept of QoL and domains affected might be quite different. For example because patients with KS are generally younger and (when stimulated by caregivers) more active than patients with dementia [5]. Development of a feasible observation scale tailored for this patient group might encourage future studies to focus on QoL in this patient group. Therefore, the aims of the current study were to develop an observational KS-specific measure for assessing QoL and to explore its scalability, reliability and construct validity.

## Method

The QUALIKO was developed in two phases. In phase 1, relevant domains and items were identified and formulated based on literature review and two surveys among an expert panel of professionals involved in the care for patients with KS. In phase 2, the preliminary version of the instrument was field tested for psychometric properties.

### Phase 1: development and selection of the dimensions and items

For developing a KS-specific QoL measure, it is important to identify those dimensions of QoL that are particularly relevant to this target group. A review of the literature revealed only three studies examining QoL in patients with KS in long-term care facilities [12–14]. All three studies used the QUALIDEM. No other studies were available that focused on the assessment or important components of QoL in KS. Therefore, potentially relevant dimensions additional to those from the QUALIDEM were identified from the literature on studies on the assessment of QoL in general [16–18], in elderly populations [19, 20], and in specific populations with severe cognitive impairments [21–23]. Categorization of these dimensions resulted in 20 dimensions potentially relevant for patients with KS (see Table 1).

**Table 1:** Dimensions selected by KS experts (*N* = 19) as most important for QoL of patients with KS

Dimension	Number of caregivers (%)^**a**^
Care relationship	18 (94.7%)
Feeling at home	16 (84.2%)
Meaningful activity	14 (73.7%)
Autonomy	13 (68.4%)
Positive self-image	13 (68.4%)
Positive affect	8 (42.1%)
Restless tense behavior	6 (31.6%)
Activities / hobbies	4 (21.1%)
Acceptance of KS and associated limitations	3 (15.8%)
Physical health	2 (10.5%)
Degree of limitations caused by KS	1 (5.3%)
Mobility	1 (5.3%)
Social relations / social support	1 (5.3%)
Work	1 (5.3%)
Symptoms of depression	0
Symptoms of anxiety	0
Sleep disturbances	0
Aggression / irritability	0
Material issues	0
Religiousness	0

^**a**^ Each KS expert was asked to select a maximum of 5 dimensions, but some caregivers selected more dimensions. *KS* Korsakoff syndrome

A short survey including brief explanations and examples of behaviors representative for each dimension was performed among an expert panel of 19 professional caregivers experienced with working with patients with KS. All caregivers were employees from Krönnenzommer, ZorgAccent (Hellendoorn, the Netherlands), a nursing home specialized in care for patients with KS. The experts were asked to select the five dimensions from the list of 20 they considered most important for the QoL of patients with KS. Five dimensions that were selected by the majority (over two-third) of the experts were identified for inclusion in the research version of the instrument: Care relationship, Feeling at Home, Meaningful activity, Autonomy, and Positive Self-Image (see Table 1). Positive Affect and Restless Tense Behavior, selected by 42 and 32% of the experts respectively, were also considered sufficiently relevant for inclusion. Although Social Relations / Social Support was selected by only one expert, this dimension was also included as social wellbeing, next to physical and psychological wellbeing, is a fundamental aspect of overall health-related QoL [16, 17] and appeared to be underrepresented in the other dimensions. Finally, like in the QUALIDEM, a separate dimension for Negative Affect was added.

Where possible, subscales and items of the QUALIDEM covering these dimensions were selected for inclusion in a preliminary QUALIKO instrument. Eight subscales and corresponding items of the QUALIDEM directly matched the selected dimensions: Care Relationship (7 items), Positive Affect (6 items), Negative Affect (3 items), Restless Tense Behavior (3 items), Positive Self-Image (3 items), Social Relations (6 items), Social Isolation (3 items), and Feeling at Home (4 items). Both Autonomy and Meaningful activity were not included in the QUALIDEM, but were considered important dimensions by the KS experts. However, the QUALIDEM does contain a subscale ‘Having Something to Do’, consisting of 2 items (‘Finds things to do without help from others’ and ‘Enjoys helping with chores on the ward’). These items were also considered relevant for Meaningful activity of KS patients. Five additional items were formulated to constitute a new Meaningful activity subscale. For Autonomy, 6 potential new items were formulated. Care was taken in formulating the items in such a way that they were applicable to patients with KS living in nursing homes.

Next, the resulting preliminary version of the QUALIKO was evaluated by the same expert panel. Each member was presented with the observation scale and was asked to judge the items based on 1) relevance for QoL, 2) formulation, 3) observability and 4) applicability to patients with KS. Experts were asked to pay particular attention to the newly developed items of the Meaningful activity and Autonomy subscales. Based on the comments and issues raised by the experts: several items were reformulated, one of the new items and 2 of the original QUALIDEM items (‘Cries’, ‘Calls out’) were removed altogether as they were not considered relevant for patients with KS, and 3 new items were added (‘Indicates to want more independence than he or she can handle’, ‘Feels safe’, and ‘Indicates to miss contact with family’).

### Phase 2: field testing

#### Participants and procedure

The 48-item QUALIKO version resulting from phase 1 was subsequently field tested for psychometric properties among patients with KS living in the Krönnenzommer nursing home. All patients fulfilled the diagnostic criteria for KS [24] and Alcohol-Induced Persisting Amnestic Disorder according to the DSM-IV-TR [25]. For each patient, the QUALIKO instrument was completed independently by two primary professional caregivers who were responsible for the daily care of the respective patients. The caregivers received the questionnaire on the same day and were instructed to complete them after a one-week observation period. All questionnaires were completed within a period of 2 weeks. Caregivers were additionally instructed to complete the instrument independently, and not to consult one another when in doubt. In total, 17 primary caregivers completed the observation scale. Primary caregivers received a brief verbal instruction on using the instrument.

In total, the QUALIKO was completed by two primary caregivers for 77 patients with KS. The majority of the patients (*N* = 61; 79.2%) were men. The mean age of the patients was 60.4 (SD = 6.9) and their average duration of stay in the nursing home was 7.2 (SD = 5.6) years. Most patients were divorced (*n* = 43; 55.8%), 20 patients (20.6%) were never married, 8 patients (10.4%) were married or living together, and 5 patients (6.6%) were widowed.

#### Instruments

The QUALIKO instrument for field testing consisted of 48 items describing observable behaviors, printed in random order. Each QUALIKO form contained a separate short written instruction on how to complete the instrument. The instruction included that the items should be scored over the past week of observing the patient. The four response options for each item were never (0), seldom (1), sometimes (2), and often (3). An explanation was provided for the different response options: never (never in the past week), rarely (at most once in the past week), sometimes (a few times in the past week), and often (almost all days). Twenty-five items were positively formulated (e.g., ‘Appreciates help that he or she receives’) and 23 items were negatively formulated (e.g., ‘Rejects help from nursing assistant’). Responses for the 23 negatively formulated QUALIKO items were recoded so that higher scores indicated better QoL. Besides the QUALIKO, two primary caregivers and their respective team supervisor (e.g., the head nurse) judged the global QoL of each patient on a 10-point numerical rating scale ranging from “very poor” (1) to “very good” (10).

#### Analysis

Feasibility or practicality of completing the QUALIKO was determined from the percentage of missing values for individual items.

Scalability of the assumed subscales of the QUALIKO was examined separately for both observers using Mokken scale analysis [26]. Mokken scale analysis is a nonparametric item response theory-based method for constructing unidimensional sets of items and is ideally suited when the intention is to score an underlying latent trait by simple summation of the item response values [27]. The evaluation of a scale using Mokken scaling results in Loevinger’s coefficient *H* as an indicator for the scalability of each item and subscale. The scalability of a single item in relation to the other items in the scale or an item set is expressed by the value *H*_i_. For an item to be coherent enough to be included in a unidimensional subscale, its *H*_i_ value should be > 0.30 [27, 28]. Therefore, items with *H*_i_ values ≤0.30 in either or both observers were considered not scalable and iteratively deleted from the subscale, starting with the item with the lowest *H*_i_ coefficient. The scalability of the total subscale is expressed by *H*^T^, summarizing the accuracy of item ordering within a scale. Total subscales should have a *H*^T^ value of at least 0.30 to form a weak scale. Values of *H*^T^ between 0.40 and 0.50 indicate moderate scalability and *H*^T^ values of 0.50 and above indicate strong scalability [29].

The reliability of each subscale was estimated for both observers by calculating Molenaar-Sijtsma’s Mokken’s rho (*ρ*) coefficients. Comparisons of Mokken’s *ρ* and the classical reliability estimate Cronbach’s alpha (*α*) showed that Mokken’s *ρ* mostly led to only slightly biased approximations of the true reliability. Furthermore, they were always less biased than coefficient *α* [30, 31]. Coefficients ≥0.70, ≥0.80 and ≥ 0.90 are generally considered to indicate acceptable, good and excellent reliability, respectively [32]. For comparison purposes, we also calculated Cronbach’s *α* internal consistency coefficients for each subscale. To examine whether scores can be generalized across observers, interobserver reliability of the subscales was calculated by computing intraclass correlation coefficients (ICCs) with 95% confidence intervals (CIs) using two-way random effects models with absolute agreement for single measurements (type A,1) [33]. According to Fleiss, ICCs < 0.40 indicate poor agreement, between 0.40 to 0.75 fair to good agreement and values > 0.75 excellent agreement [34]. Additionally, quadratic weighted kappa (*Κ*_*w*_) coefficients with 95% asymptotic CIs for categorical data were computed to estimate the interobserver reliability of the individual items [35]. *Κ*_*w*_ values for individual items between 0.21 to 0.40 were considered to indicate a fair strength of agreement, between 0.41 to 0.60 moderate agreement and between 0.61 to 0.80 substantial agreement [36].

For examining the construct validity of the QUALIDEM, summed subscale scores for the remaining items were computed for both observers and averaged by taking the arithmetic mean. To assess the internal construct validity of the QUALIKO, Pearson correlation coefficients (*r*’s) were computed between the subscale scores. As QoL is considered to be a multidimensional construct it was hypothesized that correlations between the subscales would be at most moderate (≤0.70) [37]. High correlations (> 0.70) were considered undesirable because this would question the distinctiveness of the subscales. For external construct validity (convergent validity), Pearson correlations were computed with the global ratings of QoL made by the supervisors and primary caregivers. All subscale scores were expected to demonstrate significant weak to moderate (*r* = 0.10 to 0.69) positive associations with global ratings of QoL.

For exploring the interpretability, mean scores and standard deviations (SDs) were computed for the subscales. Potential associations of QUALIDEM subscale scores with age and differences in scores between sexes were explored using Pearson correlation coefficients and independent-sample *t*-tests, respectively. Additionally, floor effects (proportion of patients scores the lowest, worst possible score) and ceiling effects (proportion of patients scores the highest, best possible score) of the subscale scores were examined. If a large proportion of patients scores the minimum or maximum possible value on a subscale, this may point to limited content validity and result in reduced reliability and responsiveness to either deteriorations or improvements in QoL [38, 39].

Mokken scale analysis was performed with the Mokken package version 2.8.11 in R [40]. All other analyses were performed using SPSS version 25.

## Results

### Missing value analysis

Of the 48 initial QUALIKO items, only 31 responses (0.4%) from a maximum of 7392 possible responses were missing across the two observers (17 responses for primary caregiver 1 vs. 14 for primary caregiver 2), suggesting completing the instrument was feasible for the primary caregivers. Across the two observer groups, the proportion of missing values ranged from 0% for 36 items to 3.9% for 2 items (‘Is productive at day care’ and ‘Complains about day care’). Missing item responses were imputed using the expectation maximization algorithm [41], which has been shown to produce more accurate estimates than other methods such as deletion of missing cases or mean substitution [42], and rounded to the nearest integer for subsequent analyses.

### Item analysis

Individual item-response distributions tended to be negatively skewed, with more patients scoring at a higher level of QoL, confirming the applicability of non-parametric scaling analysis. For twelve items, not all item-response options were used by either or both observer groups. In all these cases, the most negative response option (‘never’ or ‘always’) was not used for any patient.

### Scalability

Item scalability coefficients were generally comparable between observer groups. Six items from 4 subscales had scalability coefficients below 0.30 and were deleted from their respective subscale for subsequent analysis. Five of these items demonstrated poor scalability in both observers, while 1 item demonstrated poor scalability in one observer group and borderline scalability in the other. Two items from the subscale Autonomy were deleted, namely ‘Is capable of taking care of themselves’ (Caregiver 1: *H*_i_ = − 0.09; Caregiver 2: *H*_i_ = − 0.22) and ‘Makes independent choices when possible’ (Caregiver 1: *H*_i_ = − 0.39; Caregiver 2: *H*_i_ = − 0.05). Two items were also deleted from the Social relations subscale, namely ‘Cuts himself/herself off from environment’ (Caregiver 1: *H*_i_ = 0.15; Caregiver 2: *H*_i_ = 0.14) and ‘Feels at ease in the company of others’ (Caregiver 1: *H*_i_ = 0.30; Caregiver 2: *H*_i_ = 0.23). Additionally, the item ‘Indicates feeling worthless’ (Caregiver 1: *H*_i_ = − 0.003; Caregiver 2: *H*_i_ = 0.015) from the positive self-image subscale and ‘Complains about day care’ (Caregiver 1: *H*_i_ = − 0.10; Caregiver 2: *H*_i_ = 0.04) from the Productive subscale were removed because of poor scalability.

The scalability coefficients of the remaining 42 items are presented in Table 2. Except for Positive self-image, total *H*^T^ values indicated at least moderate scalability of the subscales in both observer groups. Four subscales (Care relationship, Autonomy, Positive affect, Social relations) showed strong scalability in both observer groups. Restless tense behavior, Social isolation, Feeling at home and Meaningful activity showed strong scalability in one observer group and moderate scalability in the other. Negative affect, with only two remaining items, demonstrated moderate scalability in both observer groups. Finally, the other 2-item subscale Positive self-image showed strong scalability in one observer group, but weak scalability in the other.

**Table 2:** Scalability, internal consistency and inter-rater reliability of the QUALIKO items and subscales

	Primary caregiver 1			Primary caregiver 2			
	*H*^T^ / *H*_i_	*ρ*	*α*	*H*^T^ / *H*_i_	*ρ*	*α*	ICC (95% CI) / *Κ*_*w*_ (95% CI)
**A: Care relationship**	**0.58**	**0.88**	**0.87**	**0.53**	**0.86**	**0.85**	**0.88 (0.81–0.92)**
Rejects help from caregiver	0.62			0.53			0.76 (0.65–0.87)
Is unfriendly in contacts with caregiver (is angry)	0.65			0.61			0.77 (0.67–0.86)
Has conflicts with caregiver	0.69			0.62			0.80 (0.70–0.90)
Accuses others	0.46			0.48			0.69 (0.53–0.86)
Appreciates help that he or she receives	0.45			0.39			0.67 (0.51–0.84)
Accepts help	0.50			0.51			0.78 (0.64–0.92)
Criticizes the daily routine	0.59			0.53			0.75 (0.63–0.87)
**B: Autonomy**	**0.71**	**0.90**	**0.79**	**0.65**	**0.87**	**0.77**	**0.79 (0.68–0.86)**
Indicates to want more independence than he or she can handle	0.77			0.69			0.67 (0.50–0.83)
Follows directions of caregiver	0.63			0.53			0.29 (0.05–0.52)
Indicates to want more say about his/her own life than he or she can live up to	0.74			0.72			0.74 (0.59–0.89)
Accepts the rules of the living community	0.63			0.56			0.68 (0.48–0.87)
**C: Positive affect**	**0.63**	**0.88**	**0.89**	**0.51**	**0.84**	**0.84**	**0.75 (0.64–0.84)**
Has a contented appearance	0.66			0.62			0.68 (0.51–0.85)
Smiles	0.66			0.64			0.57 (0.41–0.74)
Mood can be influenced in a positive sense	0.49			0.21			0.33 (0.10–0.57)
Is cheerful	0.71			0.66			0.63 (0.49–0.78)
Feels safe	0.52			0.52			0.67 (0.46–0.88)
Is in a good mood	0.70			0.59			0.71 (0.54–0.89)
Is capable of enjoying things in daily life	0.64			0.38			0.69 (0.53–0.85)
**D: Negative affect**	**0.47**	**0.62**	**0.58**	**0.49**	**0.64**	**0.62**	**0.72 (0.60–0.82)**
Makes an anxious impression	0.47			0.49			0.71 (0.56–0.86)
Is sad	0.47			0.49			0.70 (0.56–0.84)
**E: Restless tense behavior**	**0.47**	**0.70**	**0.70**	**0.65**	**0.82**	**0.81**	**0.67 (0.52–0.78)**
Makes restless movements	0.45			0.64			0.52 (0.31–0.72)
Is restless	0.56			0.73			0.60 (0.43–0.77)
Has tense body language	0.40			0.56			0.73 (0.62–0.84)
**F: Positive self-image**	**0.32**	**0.47**	**0.45**	**0.58**	**0.71**	**0.67**	**0.59 (0.42–0.72)**
Asks for more help than needed	0.32			0.58			0.58 (0.33–0.83)
Indicates not being able to do anything	0.32			0.58			0.51 (0.16–0.85)
**G: Social relations**	**0.69**	**0.88**	**0.78**	**0.64**	**0.84**	**0.78**	**0.89 (0.83–0.93)**
Has contact with other residents	0.76			0.74			0.75 (0.63–0.87)
Responds positively when approached	0.46			0.58			0.62 (0.51–0.84)
Takes care of other residents	0.70			0.58			0.75 (0.64–0.87)
Is on friendly terms with one or more residents	0.72			0.65			0.75 (0.60–0.89)
**H: Social isolation**	**0.47**	**0.70**	**0.70**	**0.65**	**0.82**	**0.81**	**0.62 (0.46–0.74)**
Openly rejects contact with others	0.45			0.64			0.59 (0.39–0.78)
Is rejected by other residents	0.56			0.73			0.64 (0.52–0.76)
Indicates to miss contact with family	0.40			0.56			0.69 (0.54–0.84)
**I: Feeling at home**	**0.52**	**0.78**	**0.78**	**0.49**	**0.80**	**0.77**	**0.69 (0.55–0.79)**
Indicates that he or she is bored	0.36			0.39			0.44 (0.21–0.68)
Indicates feeling locked up	0.62			0.56			0.70 (0.54–0.87)
Wants to get off the ward	0.58			0.57			0.68 (0.51–0.84)
Feels at home on the ward	0.51			0.43			0.74 (0.57–0.90)
**J: Meaningful activity**	**0.51**	**0.81**	**0.79**	**0.43**	**0.78**	**0.73**	**0.81 (0.72–0.88)**
Enjoys going to day care	0.54			0.41			0.77 (0.56–0.99)
Is productive at day care	0.69			0.55			0.53 (0.26–0.79)
Participates in activities in free time	0.42			0.37			0.74 (0.62–0.85)
Enjoys helping with chores in the living community	0.50			0.42			0.75 (0.64–0.87)
Has fun at the day care	0.57			0.47			0.58 (0.32–0.85)
Finds things to do without help from others	0.41			0.42			0.83 (0.74–0.93)

*H*^T^ = Loevinger’s scalability coefficient for the subscale; *H*_i_ = Loevinger’s scalability coefficient for the item; *ρ* = Sijtsma and Molenaar’s rho; α = Cronbach’s alpha; ICC = intraclass correlation coefficient (two-way random effects model, single measures, absolute agreement); *Κ*_*w*_ = quadratic weighted kappa; CI = confidence interval

### Reliability

Reliability coefficients *ρ* indicated acceptable (≥0.70) to good (≥0.80) reliability for all subscales in both observer groups, except for the two 2-item subscales of Negative affect and Positive self-image. As would be expected, Cronbach’s *α* lower bound estimates tended to be slightly lower than Molenaar-Sijtsma *ρ* values. The results are presented in Table 2.

Inter-observer agreement was excellent (ICC > 0.75) for five subscales (Care relationship, Autonomy, Positive affect, Social relations, and Meaningful activity) and fair to moderate (ICC = 0.40 to 0.75) for the other five subscales (Negative affect, Restless tense behavior, Positive self-image, Social isolation, and Feeling at home). Inter-observer agreement for individual items was substantial (33 items) or moderate (13 items) for all except two items (‘Follows directions of nursing assistant’ and ‘Mood can be influenced in a positive sense’).

### Construct validity

Table 3 presents the Pearson correlations between the ten subscales of the QUALIKO. Correlations between subscales were mostly negligible to moderate, confirming the multidimensional structure of the QUALIKO instrument. However, two subscale intercorrelations exceeded the selected threshold for strong correlation (Care relation vs. Autonomy and Autonomy vs. Feeling at home), indicating a high degree of overlap between these subscales.

**Table 3:** Mean scores and Pearson intercorrelations of the QUALIKO subscales

	Range	Mean (SD)	% floor	% ceiling	A	B	C	D	E	F	G	H	I
A: Care relationship	0–21	15.42 (4.54)	0	9.1	–								
B: Autonomy	0–12	9.44 (2.62)	0	28.6	0.73***	–							
C: Positive affect	0–21	17.03 (3.38)	0	11.7	0.46***	0.26*	–						
D: Negative affect	0–6	4.47 (1.44)	1.3	22.1	0.42***	0.31**	0.56***	–					
E: Restless tense behavior	0–9	5.67 (2.38)	2.6	10.4	0.12	0.02	0.41***	0.30**	–				
F: Positive self-image	0–6	5.27 (1.17)	1.3	53.2	−0.11	−0.15	0.00	0.14	−0.13	–			
G: Social relations	0–12	8.16 (2.99)	0	9.1	−0.09	− 0.26*	0.43***	0.01	0.25*	−0.05	–		
H: Social isolation	0–9	6.64 (1.78)	0	13.0	0.69***	0.68***	0.34**	0.51***	0.08	0.05	−0.19	–	
I: Feeling at home	0–12	9.61 (2.60)	0	26.0	0.70***	0.78***	0.39***	0.45***	−0.05	0.03	−0.20	0.63***	–
J: Meaningful activity	0–18	12.64 (3.88)	0	3.9	0.07	−0.09	0.49***	0.16	0.13	0.19	0.69***	−0.13	0.09

* *p* < 0.05; ** *p* < 0.01; *** *p* < 0.001, two-sided

With the exception of Positive self-image, all subscales of the QUALIKO were significantly and positivity correlated with the primary caregivers’ global ratings of QoL (Table 4). Correlation coefficients were all in the range of weak to moderate and were strongest for Positive affect, Negative affect and Meaningful activity. Interestingly, global QoL ratings by the respective team supervisors were not significantly correlated with Autonomy, Positive self-image, Social isolation, and Feeling at home.

**Table 4:** Pearson correlations of the QUALIKO subscales with global ratings of QoL made by the team supervisor and primary caregivers

	Team manager (*N* = 77)	Primary caregiver 1 (*N* = 77)	Primary caregiver 2 (*N* = 77)
Team supervisor			
Primary caregiver 1	0.73***		
Primary caregiver 2	0.75***	0.62***	
A: Care relationship	0.32**	0.43***	0.34**
B: Autonomy	0.10	0.26*	0.23*
C: Positive affect	0.63***	0.69***	0.60***
D: Negative affect	0.34**	0.56***	0.46***
E: Restless tense behavior	0.48***	0.40***	0.36**
F: Positive self-image	0.09	0.11	0.14
G: Social relations	0.49***	0.32**	0.32**
H: Social isolation	0.07	0.23*	0.25*
I: Feeling at home	0.21	0.36**	0.31**
J: Meaningful activity	0.68***	0.54***	0.46***

* *p* < 0.05; ** *p* < 0.01; *** *p* < 0.001, two-sided

### Interpretability

Mean scores on the QUALIKO are reported in Table 3. Floor effects were absent or negligible for all subscales. For all subscales, mean scores tended to be relatively high, but especially total scores for positive self-image were severely skewed and demonstrated a large ceiling effect. Most of the patients achieved the highest possible score for this dimension. Age was not significantly associated with any of the subscale scores (*r* values between − 0.22 and 0.21, all *p* values > 0.05). Male patients scored significantly higher on Care relationship, Positive affect, Negative affect, Social isolation, and Feeling at home compared to female patients (Table 5).

**Table 5:** Mean scores on the QUALIKO subscales stratified by the patients’ sex

	Men (*N* = 61) Mean (SD)	Women (*N* = 16) Mean (SD)	*t* (df = 75)	p*
A: Care relationship	16.13 (4.17)	12.72 (5.01)	2.79	0.007
B: Autonomy	9.60 (2.51)	8.81 (3.00)	1.07	0.289
C: Positive affect	17.56 (3.24)	15.00 (3.22)	2.81	0.006
D: Negative affect	4.81 (1.18)	3.16 (1.61)	4.60	< 0.001
E: Restless tense behavior	5.68 (2.24)	5.63 (2.95)	0.08	0.935
F: Positive self-image	5.36 (0.99)	4.94 (1.67)	1.30	0.198
G: Social relations	8.02 (2.96)	8.69 (3.14)	−0.79	0.433
H: Social isolation	6.98 (1.53)	5.34 (2.10)	3.52	< 0.001
I: Feeling at home	9.99 (2.15)	8.16 (3.58)	2.61	0.011
J: Meaningful activity	12.49 (3.92)	13.19 (3.78)	−0.64	0.527

* Two-sided

## Discussion

The aim of this study was to develop a feasible observational measure of QoL for patients with KS living in nursing homes. The study resulted in an observation scale that measures 10 aspects of QoL relevant to patients with KS: the QUALIKO. Field testing of the instrument showed that the final 42-item QUALIKO (see Appendix) demonstrated adequate to strong scalability of the subscales, except for Positive self-image. Except for Negative affect and Positive self-image, all subscales demonstrated acceptable to good internal consistency reliability and inter-observer reliability was fair to excellent for all subscales. Construct validity was supported by negligible to moderate intercorrelations between the subscales and, except for Positive self-image, weak to moderate correlations with caregivers’ global QoL ratings. Overall, our findings indicate that the QUALIKO is a feasible, reliable and valid observational measure of QoL in residential KS patients. The interpretation of the Positive self-image subscale should be done with some caution, as the psychometric properties are suboptimal.

The QUALIDEM, which was originally developed for use in patients with dementia [10, 11], turned out to provide a good basis for assessing QoL in patients with KS. In the final QUALIKO instrument, 30 items from the original 40-item QUALIDEM were retained or only slightly adapted and 8 out of its 9 subscales were retained. However, the QUALIKO also contains 12 new items and 2 additional dimensions of QoL (Autonomy and Meaningful activity) that were considered important by specialized caregivers for patients with KS. This is not surprising, as patients with KS are generally younger and more active than patients with dementia. As such, the QUALIKO may have better content validity for specific use in residential patients with KS living in nursing homes.

The field test showed that 42 items of the QUALIKO formed subscales with, in general, moderate to strong scalability. Scalability and reliability coefficients of the subscales were quite similar to those found for the QUALIDEM in different samples of patients with dementia in residential settings [43], with especially the Social relations and Social isolation subscales showing notably better scalability and reliability in the current study. Scalability and reliability were, however, lower for Negative affect and Positive self-image, possible due to the fact that only two items were retained for these subscales. Interobserver agreement was excellent for 5 subscales and fair to moderate for the other 5 subscales, suggesting that scores are sufficiently comparable between observers.

The multidimensional internal structure of the QUALIKO was confirmed by low to moderate intercorrelations between most of the subscales and, except for positive self-image, all subscales were significantly correlated with primary caregivers’ global ratings of QoL, supporting the construct validity of the instrument. Also, missing values for individual items were minimal, and comparable to those found in previous studies with the QUALIDEM (range of missing item responses: 0.2–0.4%) [43], supporting the feasibility of QUALIKO administration in the nursing home setting. Finally, floor and ceiling effects were acceptable for most subscales, suggesting that the QUALIKO is potentially responsive to measuring changes in QoL [38, 39].

However, the items of the Positive self-image dimension, which was considered an important aspect of QoL of residents with KS by the specialized caregivers, may need to be reformulated. This subscale demonstrated the lowest scalability and reliability and a notable lack of correlation with most of the other subscales and with global ratings of QoL. The content of this dimension may need to be aimed more at assessing a patient’s self-worth and self-acceptance. Lack of insight into their condition is typical of this patient group and patients themselves may believe that nothing is wrong with them [7]. The need for living in long-term care settings, and additionally losing independence and autonomy, is particularly frustrating for these patients, and might subsequently have negative effects on a patient’s self-image.

Although the current study demonstrated promising preliminary psychometric properties of the QUALKO, future studies are needed to investigate other important measurement properties the instrument. First of all, reproducibility (test-retest reliability) of QUALIKO scores needs to be examined in a stable sample of KS patients. Also, relevant normative data derived from a large and representative sample of KS patients should be established to increase the usability and interpretability of QUALIKO scores for research and daily clinical care of individual KS patients. The finding that 5 out of 10 subscale scores were significantly different between male and female patients suggests that sex-specific norms may be needed.

The major limitation of the psychometric evaluation of the QUALIKO in this study is the relatively small sample size of the field study, especially for dimensionality and scalability analysis. For instance, the sample size was considered too small for exploratory factor analysis of the dimensional structure of the instrument, which as a very general rule of thumb requires at least 5 to 10 subjects per item [44]. Although our sample of 77 KS patients is substantial compared to most published research in this patient group (due to its relatively low prevalence), much larger samples are required for thoroughly confirming item monotonicity and the absence of local response dependence and differential item functioning. Although the non-parametric Mokken analysis used in this study provides a preliminary indication of the unidimensionality and scalability of the subscales of the QUALIKO, future studies need to confirm these psychometric properties in larger samples using appropriate techniques such as confirmatory factor analysis or parametric item response theory analysis.

On the other hand, the sample size was considered sufficiently large for both reliability and construct validity analysis. For instance, the sample size of 77 observations allowed accurate estimation of excellent inter-observer agreement values (ICC > 0.75) for two raters with a small 95% confidence interval width of at most 0.2 (i.e., between 0.65 and 0.85) around the estimate and a slightly wider confidence interval width of 0.4 for fair agreement (ICC = 0.40) [45]. Furthermore, it provided around 80% power to detect even weak bivariate correlations of at least *r* = 0.30 as statistically significant at a two-tailed *α* = 0.05 level for the construct validity analyses.

Two subscales in the final QUALIKO (Negative affect and Positive self-image) ended up consisting of two items only. Although Cronbach’s *α* values were reported for these subscales, several researchers have indicated that Spearman-Brown split-half reliability is a more appropriate reliability coefficient for two-item scales [46, 47]. We decided to only report Cronbach’s *α* estimates also for these two subscales, since additional analyses (not reported) showed that these were almost identical to Spearman-Brown split-half reliability estimates for both subscales in both observer groups, suggesting that the measurement error variances are equivalent for the two items in both scales [47].

## Conclusions

This study described the development and preliminary evaluation of the first disease-specific observation scale that can be used by professional caregivers to measure the QoL in patients with KS residing in nursing homes. Development of this feasible observation scale might encourage future studies to focus on QoL in this patient group. The QUALIDEM showed promising measurement qualities, but more studies in larger groups of KS patients are needed to confirm the psychometric properties of the instrument, to collect normative data and to establish its test-retest reliability.

## Data Availability

The datasets used and/or analyzed during the current study are available from the corresponding author on reasonable request.

## **Appendix**

### English-language translation of the QUALIKO

This questionnaire contains 42 questions. Answer the questions based on your observations of the patient in the last week. Please answer every question. If you are not sure which response option is best, please select the option that most closely represents your observations. An answer is never incorrect; always select the option that resembles reality most closely from your perspective. Do not overthink your answers; the first option that comes to mind is usually the best.

Never = Never in the past week

Rarely = Once at most in the last week

Sometimes = A few times a week

Frequently = Almost daily

**Table:**

		Never	Rarely	Sometimes	Frequently	
1	Has a contented appearance	0	1	2	3	C
2	Enjoys going to day care	0	1	2	3	J
3	Has contact with other residents	0	1	2	3	G
4	Rejects help from caregiver	0	1	2	3	A
5	Makes restless movements	0	1	2	3	E
6	Is unfriendly in contacts with caregiver (is angry)	0	1	2	3	A
7	Indicates that he or she is bored	0	1	2	3	I
8	Is productive at day care	0	1	2	3	J
9	Indicates to want more independence than he or she can handle	0	1	2	3	B
10	Smiles	0	1	2	3	C
11	Asks for more help than needed	0	1	2	3	F
12	Has conflicts with caregiver	0	1	2	3	A
13	Indicates feeling locked up	0	1	2	3	I
14	Indicates not being able to do anything	0	1	2	3	F
15	Accuses others	0	1	2	3	A
16	Responds positively when approached	0	1	2	3	G
17	Appreciates help that he or she receives	0	1	2	3	A
18	Is restless	0	1	2	3	E
19	Makes an anxious impression	0	1	2	3	D
20	Takes care of other residents	0	1	2	3	G
21	Follows directions of caregiver	0	1	2	3	B
22	Wants to get off the ward	0	1	2	3	I
23	Is sad	0	1	2	3	D
24	Openly rejects contact with others	0	1	2	3	H
25	Mood can be influenced in a positive sense	0	1	2	3	C
26	Participates in activities in free time	0	1	2	3	J
27	Accepts help	0	1	2	3	H
28	Feels at home on the ward	0	1	2	3	I
29	Is cheerful	0	1	2	3	C
30	Feels safe	0	1	2	3	C
31	Is rejected by other residents	0	1	2	3	H
32	Criticizes the daily routine	0	1	2	3	A
33	Is in a good mood	0	1	2	3	C
34	Indicates to miss contact with family	0	1	2	3	H
35	Enjoys helping with chores in the living community	0	1	2	3	J
36	Is capable of enjoying things in daily life	0	1	2	3	C
37	Has tense body language	0	1	2	3	E
38	Is on friendly terms with one or more residents	0	1	2	3	G
39	Indicates to want more say about his/her own life than he or she can live up to	0	1	2	3	B
40	Accepts the rules of the living community	0	1	2	3	B
41	Has fun at the day care	0	1	2	3	J
42	Finds things to do without help from others	0	1	2	3	J

How would you rate the global quality of life of the patient on a scale from 1 to 10?

**Table:**

Very poor				Average				Very good	
1	2	3	4	5	6	7	8	9	10

Do you have additional remarks?

*Scoring:* the letter next to each question corresponds to the subscale to which this item belongs. Add the item scores per subscale.

**Table:**

	Subscale (Number of items)	Scoring range	Score
C	Positive affect (7)	0–21	
D	Negative affect (2)	0–6	
E	Restless tense behavior (3)	0–9	
F	Positive self-image (2)	0–6	
G	Social relations (4)	0–12	
H	Social isolation (3)	0–9	
I	Feeling at home (4)	0–12	
J	Meaningful activity (6)	0–18	

## Abbreviations

ICC: Intraclass correlation coefficient
KS: Korsakoff’s syndrome
QoL: Quality of life
SD: Standard deviation
CI: Confidence interval
